# Draft Genome Sequences of Lawsonia intracellularis Swine Strains Causing Proliferative Enteropathy in Japan

**DOI:** 10.1128/MRA.01021-18

**Published:** 2018-09-06

**Authors:** Sayaka Nishikawa, Yohsuke Ogawa, Masahiro Eguchi, Anura Rambukkana, Yoshihiro Shimoji

**Affiliations:** a National Institute of Animal Health, National Agriculture and Food Research Organization (NARO), Tsukuba, Ibaraki, Japan; b MRC Centre for Regenerative Medicine, Centre for Infectious Diseases, The University of Edinburgh, Edinburgh, Scotland; c Research Institute for Biomedical Sciences, Tokyo University of Science, Noda, Chiba, Japan; University of Arizona

## Abstract

The draft genome sequences of three strains of Lawsonia intracellularis, an obligate intracellular animal pathogen responsible for causing proliferative enteropathy, obtained from swine in different prefectures in Japan revealed the absence of a genomic island previously reported to be linked to host adaptation and to high genomic diversity, despite geographical proximity.

## ANNOUNCEMENT

Lawsonia intracellularis is a Gram-negative obligate intracellular bacterium that causes proliferative enteropathy (PE) in a wide range of animal species (1). Porcine PE, which is characterized by thickening of intestinal mucosa due to hyperplasia of crypt epithelia/enterocytes with inflammation in the jejunum, ileum, and colon, mainly affects growing/finishing pigs and results in substantial economic losses in the pig industry (1). So far, only three strains have been sequenced, two swine strains, PHE/MN1-00 and N343 (2), and an equine strain, E40504 (3). In this article, we report the draft genome sequences of three swine strains.

Strains Fu/JPN, Ni/JPN, and Ib2/JPN were all isolated from the thickened mucous membrane of the ileums of swine macroscopically diagnosed with PE at slaughterhouses in three different prefectures in Japan. The intestines were cut into approximately 15-cm lengths and opened longitudinally. The lumen was washed, disinfected with a 10% povidone-iodine solution, and rinsed with phosphate-buffered saline (PBS). The mucous membrane lesions were isolated, suspended in PBS, and incubated at 37°C for 30 min in PBS containing 0.05% trypsin and 0.02% EDTA. After incubation, cell debris was removed by centrifugation at 1,000 rpm for 8 min, and the supernatant was filtered through a filter paper and then centrifuged at 15,000 rpm for 15 min. The pellets were subjected to DNA extraction using the QIAamp DNA minikit (Qiagen, CA) and were confirmed to include L. intracellularis genomic DNA by PCR with the L. intracellularis-specific primers LI-No.6-F (5ʹ-TCTCAGTGAGATGGGATCATGGATGGATAG-3ʹ) and LI-No.6-R (5ʹ-GAACTTGGCTCATTAACTCGCTTACTCCAT-3ʹ), both of which were designed based on the reference genome sequence of strain PHE/MN1-00. DNA libraries were prepared using the TruSeq DNA PCR-free library prep kit (Illumina, CA) according to the manufacturer’s instructions. Sequencing was performed using an Illumina HiSeq X Ten sequencing platform (150-bp paired-end reads). Following trimming of low-quality reads with ConDeTri (4), mapping of the Illumina reads to the reference genome sequence and the single-nucleotide polymorphism (SNP) calling was performed as previously described (5).

The numbers of short reads of the Fu/JPN, Ni/JPN, and Ib2/JPN strains were 3,033,172 bp, 3,282,666 bp, and 13,115,606 bp, respectively, all of which were mapped at high sequence identity (>99.0%), resulting in 286-, 301-, and 1,172-fold sequence depths, respectively. The proportions of the short reads to the total sequence reads obtained with the pig samples were 3.70% (Fu/JPN), 3.55% (Ni/JPN), and 15.67% (Ib2/JPN). The mapped reads were extracted using SAMtools version 0.1.19 (6) and assembled using Platanus version 1.2.4 (7). In the three strains, the final assembly comprised 6 (Fu/JPN and Ni/JPN) or 7 contigs (Ib2/JPN) (>1,000 bp), with a total genome size of 1.69 Mb, a GC content of 32.9%, and an *N*_50_ value of 700 kb, as evaluated by QUAST (8). Compared to the reference genome sequence of strain PHE/MN1-00, the U.S. strain N343 has 24 SNPs, whereas strains Fu/JPN, Ni/JPN, and Ib2/JPN have 99, 456, and 552 SNPs, respectively, indicating the genetic diversity of the Japanese strains. The 18-kb genomic island previously reported to be linked to the host adaptation (9) was missing from the genomic assembly of these Japanese strains.

### Data availability.

The draft genome sequences have been deposited at DDBJ/EMBL/GenBank under the accession numbers QNHO00000000 (Fu/JPN), QNHN00000000 (Ni/JPN), and QNHM00000000 (Ib2/JPN). Raw data are available in the NCBI Sequence Read Archive (SRA) database with accession numbers DRX133711 (Fu/JPN), DRX133712 (Ni/JPN), and DRX133713 (Ib2/JPN).

## References

[1] Smith DGE, Lawson GHK 2001 *Lawsonia intracellularis*: getting inside the pathogenesis of proliferative enteropathy. Vet Microbiol 82:331–345. doi:10.1016/S0378-1135(01)00397-2. 11506927

[2] Sait M, Aitchison K, Wheelhouse N, Wilson K, Lainson FA, Longbottom D, Smith DGE 2013 Genome sequence of *Lawsonia intracellularis* strain N343, isolated from a sow with hemorrhagic proliferative enteropathy. Genome Announc 1:e00027-13. doi:10.1128/genomeA.00027-13. PMC3587925 23472224

[3] Mirajkar NS, Kelley MR, Gebhart CJ 2017 Draft genome sequence of *Lawsonia intracellularis* strain E40504, isolated from a horse diagnosed with equine proliferative enteropathy. Genome Announc 5:e00330-17. doi:10.1128/genomeA.00330-17. 28495781 PMC5427216

[4] Smeds L, Künstner A 2011 ConDeTri—a content dependent read trimmer for Illumina data. PLoS One 6:e26314. doi:10.1371/journal.pone.0026314. 22039460 PMC3198461

[5] Ogawa Y, Shiraiwa K, Ogura Y, Ooka T, Nishikawa S, Eguchi M, Hayashi T, Shimoji Y 2017 Clonal lineages of *Erysipelothrix rhusiopathiae* responsible for acute swine erysipelas in Japan identified by using genome-wide single-nucleotide polymorphism analysis. Appl Environ Microbiol 83:e00130-17. doi:10.1128/AEM.00130-17. 28314730 PMC5440707

[6] Li H, Handsaker B, Wysoker A, Fennell T, Ruan J, Homer N, Marth G, Abecasis G, Durbin R; 1000 Genome Project Data Processing Subgroup. 2009 The Sequence Alignment/Map format and SAMtools. Bioinformatics 25:2078–2079. doi:10.1093/bioinformatics/btp352. 19505943 PMC2723002

[7] Kajitani R, Toshimoto K, Noguchi H, Toyoda A, Ogura Y, Okuno M, Yabana M, Harada M, Nagayasu E, Maruyama H, Kohara Y, Fujiyama A, Hayashi T, Itoh T 2014 Efficient de novo assembly of highly heterozygous genomes from whole-genome shotgun short reads. Genome Res 24:1384–1395. doi:10.1101/gr.170720.113. 24755901 PMC4120091

[8] Gurevich A, Saveliev V, Vyahhi N, Tesler G 2013 QUAST: quality assessment tool for genome assemblies. Bioinformatics 29:1072–1075. doi:10.1093/bioinformatics/btt086. 23422339 PMC3624806

[9] Vannucci FA, Kelley MR, Gebhart CJ 2013 Comparative genome sequencing identifies a prophage-associated genomic island linked to host adaptation of *Lawsonia intracellularis* infections. Vet Res 44:49. doi:10.1186/1297-9716-44-49. 23826661 PMC3716683

